# Kératoacanthome de l’avant-bras gauche

**DOI:** 10.11604/pamj.2018.30.12.15293

**Published:** 2018-05-05

**Authors:** Youssef Zemmez, Naoufal Hjira

**Affiliations:** 1 Service de Dermatologie Hôpital Militaire d'Instruction Mohamed V, Rabat, Maroc

**Keywords:** Kératoacanthome, histologie, carcinome épidermoïde, Keratoacanthoma, histology, squamous cell carcinoma

## Abstract

We here report the case of a 54-year old woman presenting with a swelling in the left forearm occurred eight weeks before and rapidly increasing in volume. Clinical examination showed ulcero-budding painless purplish skin lesion measuring 2 cm along its longer axis (A). The patient underwent simple biopsy. Histological examination showed dyskeratosic, disorganized, hyperplastic epithelium with cytonuclear abnormalities, suggesting malignant transformation. Given the absence of infiltration in the chorion and the presence of hyperkeratosis, the diagnosis of keratoacanthoma was made. Resection of the tumor was indicated to confirm or deny this diagnosis. Histological examination revealed a protruding epithelial tumor-like lesion circumscribed by two species of lateral “bird beaks” delineating a crater filled with many layers of keratin. The crater was bordered by hyperplastic epithelium. The crater base was characterized by irregular papillomatous projections as well as by few cellular strands which seemed to shred in the underlying dermis. These were basophilic made up of cells displaying a certain degree of cytonuclear abnormalites arranged in one or two peripheral areas and, in their center, some eosinophilic, keratin, homogeneous cells, few mitoses as well as many horny globes, most often completely keratinized (B). The diagnosis of keratoacanthoma was retained. Keratoacanthoma is a well defined anatomo-clinical entity which can be very difficult to distinguish from squamous cell carcinoma, whose incidence is three times higher. A distinction between these two lesions is necessary due to their different management.

## Image en médecine

Une femme âgée de 54 ans ayant consulté pour une tuméfaction de l'avant-bras gauche apparaissant huit semaines auparavant et augmentant rapidement de volume. L'examen clinique retrouvait une lésion cutanée ulcéro-bourgeonnante de 2cm de grand axe, indolore et de couleur violacée (A). Une simple biopsie a été réalisée. L'examen histologique avait montré un épithélium hyperplasique dyskératosique et désorganisé avec présence d'atypies cytonucléaires évoquant un processus tumoral malin mais vu l'absence d'infiltration du chorion et la présence d'une hyperkératose, le diagnostic d'un kératoacanthome a été soulevé et la réalisation d'une exérèse de la tumeur a été indiquée pour confirmer ou infirmer ce diagnostic. L'étude histologique révélait une lésion tumorale saillante de nature épithéliale circonscrite par deux espèces de becs latéraux limitant un cratère surmonté d'importantes strates de kératine. L'épithélium bordant ce cratère est hyperplasique. La base du cratère comporte des projections papillomateuse inégales ainsi que quelques travées cellulaires qui semblent effilocher dans le derme sous-jacent. Celles-ci sont composées de cellules basophiles montrant un certain degré d'anomalies cytonucléaires disposées en une ou deux assises périphériques et en leur centre de cellules éosinophiles, kératinisées, d'aspect homogène, quelques mitoses ainsi que de nombreux globes cornés le plus souvent complètement kératinisés (B). Le diagnostic de keratoacanthome a été retenu. Le kératoacanthome est une entité anatomo-clinique bien définie, mais qui peut être très difficile à distinguer du carcinome épidermoïde, dont l'incidence est trois fois supérieure. La distinction entre ces deux lésions est nécessaire car la prise en charge est différente.

**Figure 1:**
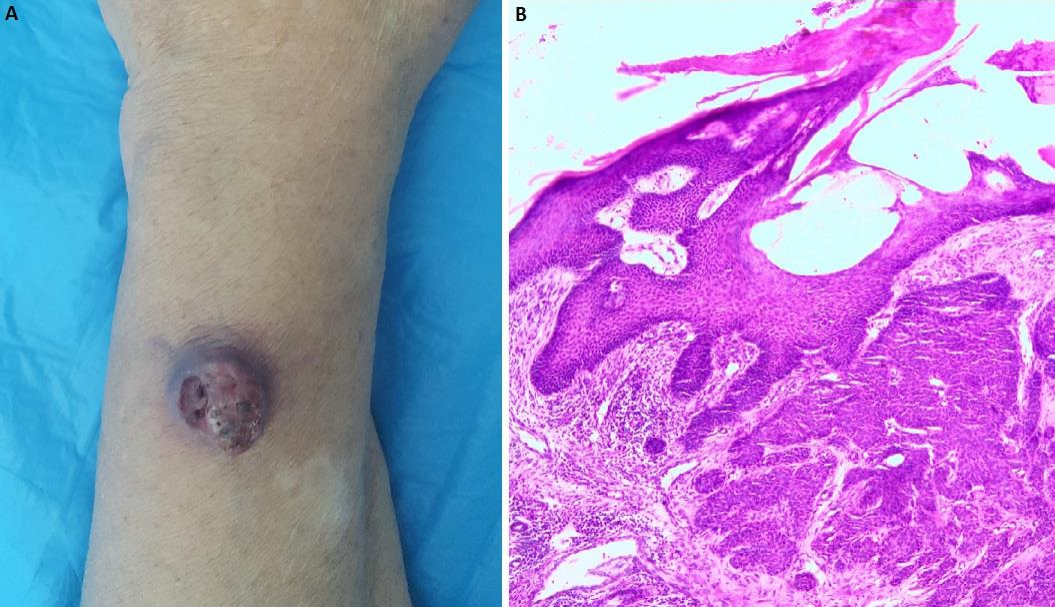
(A) lésion ulcéro-bourgeonnante de l’avant-bras gauche; (B) histologie en faveur d’un kératoacanthome

